# Lung ultrasound B-lines and serum KL-6 in the management of connective tissue disease-associated interstitial lung disease

**DOI:** 10.1515/rir-2026-0020

**Published:** 2026-10-22

**Authors:** Meigan Huang, Shaoyu Zheng, Guangzhou Du, Zexuan Zhou, Xinyi Li, Yanlin Chen, Qingzi Chen, Shijian Hu, Jianqun Lin, Kedi Zheng, Weijin Zhang, Qisheng Lin, Barbara Ruaro, Anna-Maria Hoffmann-Vold, Marco Matucci-Cerinic, Daniel E Furst, Shaoqi Chen, Yukai Wang

**Affiliations:** Department of Clinical Laboratory, Shantou Central Hospital, Shantou, Guangdong Province, China; Shantou University Medical College, Shantou, Guangdong Province, China; Department of Rheumatology and Immunology, Shantou Central Hospital, Shantou, Guangdong Province, China; Department of Radiology, Shantou Central Hospital, Shantou, Guangdong Province, China; Department of Ultrasound, The First Affiliated Hospital of Shantou University Medical College, Shantou, Guangdong Province, China; Department of Pulmonology, Cattinara Hospital, University of Trieste, Trieste, Italy; Department of Rheumatology, Oslo University Hospital, Oslo, Norway; Unit of Immunology, Rheumatology, Allergy and Rare diseases (UnIRAR), IRCCS San Raffaele Hospital, Milan, Italy; Vita Salute San Raffaele University, Milano, Italy; Division of Rheumatology, Department of Medicine, University of California at Los Angeles, Los Angeles, USA; University of Washington, Seattle, WA, USA; Department of Experimental and Clinical Medicine, Division of Rheumatology, Careggi University Hospital, University of Florence, Florence, Italy

**Keywords:** connective tissue disease, interstitial lung disease, high-resolution computed tomography, lung ultrasound, B-lines, KL-6

## Abstract

**Background and Objectives:**

Interstitial lung disease (ILD) is a major cause of morbidity and mortality in patients with connective tissue diseases (CTDs). Early diagnosis, appropriate intervention, and close monitoring are critical to mitigate the progression of CTD-ILD. Lung ultrasound (LUS) B-lines and serum Krebs von den Lungen-6 (KL-6) have emerged as promising, non-invasive, and radiation-free biomarkers for ILD. This single-center retrospective study aimed to evaluate the combined diagnostic and assessment value of B-lines and KL-6 in patients with CTD-ILD.

**Methods:**

A total of 226 consecutive patients with CTD who underwent high resolution computed tomography (HRCT), LUS and serum KL-6 testing within a one-month interval were retrospectively enrolled. Based on HRCT findings, patients were classified into ILD and non-ILD groups. Those with ILD were further subdivided into fibrotic ILD (F-ILD) and non-fibrotic ILD (NF-ILD) according to radiographic patterns. LUS was performed across 50 scanning sites, and the total number of B-lines was calculated as a B-lines number. Correlations between B-lines number, KL-6 level, and HRCT Warrick score were analyzed. The diagnostic accuracy of LUS B-lines and KL-6, individually and in combination, for detecting ILD and F-ILD was determined using receiver operating characteristic (ROC) curve analysis. The ability of B-lines and KL-6 to discriminate ILD severity based on the HRCT Warrick score was assessed.

**Results:**

The ILD group demonstrated significantly higher B-lines number and serum KL-6 levels compared to the non-ILD group (*P* < 0.001). ROC analysis showed good diagnostic performance for both B-lines (AUC = 0.85 [95% CI: 0.80–0.90]; sensitivity = 77.6%; specificity = 77.0%) and KL-6 (AUC = 0.79 [95% CI: 0.73–0.85]; sensitivity = 58.6%; specificity = 91.9%). The combination of both markers further improved diagnostic accuracy (AUC = 0.87 [95% CI: 0.82–0.91]; sensitivity = 74.3%; specificity = 86.5%). Both B-lines number and KL-6 level showed significant positive correlations with the HRCT Warrick score. Notably, B-lines number and serum KL-6 were able to distinguish between F-ILD and NF-ILD subtypes (both *P* < 0.001) and were associated with disease severity (*P* < 0.001 for B-lines; *P* = 0.016 for KL-6).

**Conclusion:**

Both LUS B-lines and serum KL-6 are valuable biomarkers for diagnosing and assessing CTD-ILD, and their combination enhances diagnostic accuracy.

## Introduction

Interstitial lung disease (ILD) is a common and severe pulmonary complication of various connective tissue diseases (CTDs), most frequently observed in rheumatoid arthritis (RA), systemic sclerosis (SSc), mixed connective tissue disease (MCTD), and idiopathic inflammatory myositis (IIM).^[1,2]^ CTD-ILD significantly impairs patients‘ quality of life and prognosis and is a major cause of mortality. High-resolution computed tomography (HRCT) is the current gold standard imaging tool for evaluating ILD, as it can detect subtle structural changes in the lung parenchyma.^[3,4]^ However, its high cost, associated radiation exposure, and unsuitability for frequent repetition limit its widespread use in the clinical practice.^[5]^ Furthermore, subclinical or asymptomatic CTD-ILD is not uncommon and can occur and persist within the first 3 to 5 years of the disease course.^[6]^ Therefore, developing sensitive, non-invasive, and repeatable diagnostic modalities is of great importance.

Lung ultrasound (LUS) was first employed for diagnosing alveolar-interstitial syndrome through the detection of comet-tail artifact in intensive care unit.^[7]^ Subsequently, LUS has been increasingly utilized in the evaluation of various diffuse parenchyma lung disease (DPLD), including CTD-ILD.^[8]^ One of its characteristic signs, B-lines, is a key sonographic pattern that reflects pulmonary interstitial abnormalities and is closely associated with alveolar septal thickening, interstitial edema, or fibrosis.^[9,10]^ Multiple studies have demonstrated a significant correlation between the number of B-lines and the extent of interstitial changes assessed by HRCT, suggesting its potential as a tool for ILD screening and monitoring.^[11,12]^ Concurrently, serum Krebs von den Lungen-6 (KL-6), a mucin-like glycoprotein released by damaged type II alveolar epithelial cells, is widely used to evaluate the activity and prognosis of interstitial lung diseases.^[13,14]^ The KL-6 assay is simple, non-invasive, and allows for dynamic monitoring, showing good diagnostic performance in various types of ILD.^[15]^

It is speculated that B-lines and KL-6 may reflect the pathophysiology of CTD-ILD at different levels: the former more directly indicates structural abnormalities of the pulmonary interstitium, whereas the latter primarily reflects biological changes related to alveolar epithelial injury and fibrosis.^[13,16]^ Accordingly, combining these two biomarkers could enhance diagnostic efficacy and the accuracy.^[17, 18, 19]^ This study aimed to assess the clinical utility of LUS B-lines and serum KL-6, individually and in combination, in managing patients with CTD-ILD.

## Materials and Methods

### Patients

A total of 226 consecutive patients with CTD who underwent HRCT, LUS, and serum KL-6 testing within a one-month interval were enrolled from the outpatient and inpatient departments of Shantou Central Hospital between January 2020 and January 2024. The enrolled patients included those with rheumatoid arthritis (RA, 114 cases), idiopathic inflammatory myopathies (IIM, 48 cases), Sjögren‘s disease (SjD, 18 cases), systemic lupus erythematosus (SLE, 17 cases), systemic sclerosis (SSc, 11 cases), mixed connective tissue disease (MCTD, 11 cases), and undifferentiated connective tissue disease (UCTD, 7 cases). The presence and patterns of ILD were defined by HRCT findings assessed by two experienced radiologists who were blind to the clinical information. Patients with a history of asthma, chronic obstructive pulmonary disease, pulmonary neoplasm, occupational or radiation-related lung disease, heart failure, renal failure, pregnancy, or other overlapping rheumatic diseases were excluded. The study was approved by the Ethics Committee of Shantou Central Hospital (No. KY-2024–018), and all procedures were conducted in accordance with the Declaration of Helsinki. Written informed consent was obtained from all participants.

### Chest High-resolution Computed Tomography

Chest HRCT scans were performed using a dual-source 128-section multi-detector CT system (Somatom Definition Flash; Siemens Healthcare, Forchheim, Germany). Scans covered the pulmonary apex to the costophrenic angle, acquired during a breath-hold at end-inspiration. Key parameters included: sequential mode, 1 mm collimation, 10 mm interval, 120 kV tube voltage, and 110 reference mAs. Images were reconstructed with an edge-enhancing B70f kernel *via* filtered back projection for lung window clinical reading. No intravenous contrast agent was used. Acquisition time was 35–45 seconds. Images had a 512×512 matrix, and effective radiation dose ranged from 3–5 mSv. The severity and extent of ILD were assessed using the Warrick score.^[20]^ The ILD group was further divided into fibrotic ILD (F-ILD) or non-fibrotic disease (NF-ILD) according to the radiographic patterns including honeycombing, traction bronchiectasis, and/ or volume loss.^[21]^ In addition, patients with ILD were stratified into mild (0–7), moderate (8–15), and severe (> 15) groups based on HRCT Warrick score ranges.^[22,23]^

### Lung Ultrasound

LUS was performed using a commercially available device (ACUSON SC2000, Siemens Medical Solutions, Erlangen, Germany) equipped with a 2.5–3.5 MHz cardiac sector transducer. In accordance with previously described methods,^[24,25]^ two senior sonographers, blinded to all clinical and HRCT data, scanned a total 50 intercostal spaces across the anterior, lateral, and posterior chest walls. The number of B-lines was recorded at each site, and the summated count yielded a semi-quantitative score reflecting the extent of ILD.^[24,26]^ A B-line is defined as a discrete, laser-like, vertical hyperechoic artifact arising from the pleural line, which extending to the bottom of the screen without fading, and moving synchronously with respiration.^[27]^

### Measurement of KL-6 Serum

KL-6 concentration (U/mL) was measured with chemiluminescent enzyme immunoassay method (LUMIPULSE G2100, Japan) in the study population.

### Statistical Analysis

All analyses were performed with SPSS version 24.0 (IBM Corp., Armonk, NY, USA). Continuous variables were compared using the *t* test or Mann-Whitney *U* test, and categorical variables were analyzed with the *chi-square* or Fisher‘s exact test. Differences among the mild, moderate, and severe groups were analyzed using the Kruskal–Wallis H test, followed by pairwise comparisons with Bonferroni-corrected. Correlations among B-lines, KL-6, and Warrick score were assessed using Spearman‘s rank correlation. Receiver Operating Characteristic (ROC) curves were generated in GraphPad Prism 10.0 (GraphPad Software, San Diego, CA, USA), and differences in area under the receiver operating characteristic curve (AUC) were compared using the DeLong test in MedCalc version 20.0 (MedCalc Software Ltd., Ostend, Belgium). Optimal cut-off values were determined by maximizing the Youden index (sensitivity + specificity − 1). A two-tailed *P* value < 0.05 was considered statistically significant.

## Results

### Baseline Characteristics of the Study Population

A total of 226 patients with CTD were included, of whom 152 patients had ILD and 74 did not. Patients in the ILD group were significantly older than those in the non-ILD group (57 [50–66] *vs*. 52 [39–56] years, *P* < 0.001). No significant differences were observed between the groups in sex distribution (female: 76.3% *vs*. 85.1%), body mass index (22.3 ± 3.4 *vs*. 22.0 ± 3.1 kg/m^2^), disease duration (12 [3–60] *vs*. 12 [3–63] months), or smoking history (19.7% *vs*. 14.9%, all *P* > 0.05).

ILD patients exhibited significantly higher median B-lines number (63 [24–150] *vs*. 10 [4–22], *P* < 0.001) and serum KL-6 levels (389.5 [251.0–781.8] *vs*. 208.9 [143.6–289.1] U/mL, *P* < 0.001) compared to the non-ILD group. In contrast, inflammatory markers, including erythrocyte sedimentation rate (ESR) (34 [17–54] *vs*. 37 [20–65] mm/h) and C-reactive protein (CRP) (8.54 [3.17–20.55] *vs*. 14.90 [4.32–43.33] mg/L) did not differ significantly between groups (both *P* > 0.05; Table 1).

**Table 1:** Baseline demographic, clinical and radiographic characteristics of the patients

Baseline characteristics	All patients (*N* = 226)	Patients (*n* = 152) with radiographic ILD	Patients (*n* = 74) without radiographic ILD	*P*
Gender, *n* (%)				0.125
Female	179 (79.2)	116 (76.3)	63 (85.1)	
Male	47 (20.8)	36 (23.7)	11 (14.9)	
Age, years, median (IQR)	55 (46–63)	57 (50–66)	52 (39–56)	<0.001
BMI, kg/m^2^,mean ± SD	22.2 (3.3)	22.3 (3.4)	22.0 (3.1)	0.526
Disease duration, months, median (IQR)	12 (3–60)	12 (3–60)	12 (3–63)	0.606
Smoking history, *n* (%)				0.372
Yes	41 (18.1)	30 (19.7)	11 (14.9)	
No	185 (81.9)	122 (80.3)	63 (85.1)	
ESR, mm/h, median (IQR)	35 (18–55)	34 (17–54)	37 (20–65)	0.373
CRP, mg/L, median (IQR)	9.67 (3.20–25.25)	8.54 (3.17–20.55)	14.9 (4.32–43.33)	0.050
B-lines number, median (IQR)	33 (11–109)	63 (24–150)	10 (4–22)	<0.001
KL-6, U/mL, median (IQR)	306.0 (202.7–525.3)	389.5 (251.0–781.8)	208.9 (143.6–289.1)	<0.001
Underlying CTD diagnoses				NA
RA, *n* (%)	114(50.4)	69(45.4)	45(60.8)	
IIM, *n* (%)	48(21.2)	39(25.7)	9(12.2)	
SjD, *n* (%)	18(8.0)	11(7.2)	7(9.5)	
SLE, *n* (%)	17(7.5)	13(8.6)	4(5.4)	
SSc, *n* (%)	11(4.9)	8(5.3)	3(4.1)	
MCTD, *n* (%)	11(4.9)	7(4.6)	4(5.4)	
UCTD, *n* (%)	7(3.1)	5(3.3)	2(2.7)	
HRCT patterns				NA
UIP, *n* (%)	19 (8.4)	19 (12.5)	0 (0)	
NSIP, *n* (%)	112 (49.6)	112 (73.6)	0 (0)	
OP, *n* (%)	15 (6.6)	15 (9.9)	0 (0)	
LIP, *n* (%)	6 (2.7)	6 (3.9)	0 (0)	
Fibrotic status				NA
Fibrotic ILD, *n* (%)		60 (39.5)		

BMI: body mass index; CRP: C-reactive protein; ESR: erythrocyte sedimentation rate; HRCT: high-resolution computed tomography; IIM: idiopathic inflammatory myopathies; ILD: interstitial lung disease; IQR: interquartile range; KL-6: Krebs von den Lungen-6; LIP: lymphocytic interstitial pneumonia; MCTD: mixed connective tissue disease; NSIP: nonspecific interstitial pneumonia; OP: organizing pneumonia; RA: rheumatoid arthritis; SD: standard deviation; SjD: Sjögren’s disease; SLE: systemic lupus erythematosus; SSc: systemic sclerosis; UCTD: undifferentiated connective tissue disease; UIP: usual interstitial pneumonia.

### Diagnostic Performance of LUS B-lines and KL-6

ROC curve analysis demonstrated that both B-lines and KL-6 exhibited favorable diagnostic performance for identifying CTD-ILD. The AUC for B-lines was 0.85 (95% CI: 0.80–0.90; Figure 1A). At an optimal cut-off value of 23, B-lines achieved a sensitivity of 77.6% and a specificity of 77.0%. For KL-6, the AUC was 0.79 (95% CI: 0.73–0.85; Figure 1B), with a sensitivity of 58.6 % and a specificity of 91.9 %, at a cut-off value of 348.7 U/mL. A combined assessment using both biomarkers further enhanced diagnostic accuracy, yielding an AUC of 0.87 (95% CI: 0.82–0.91; Figure 1C).

**Figure 1:**
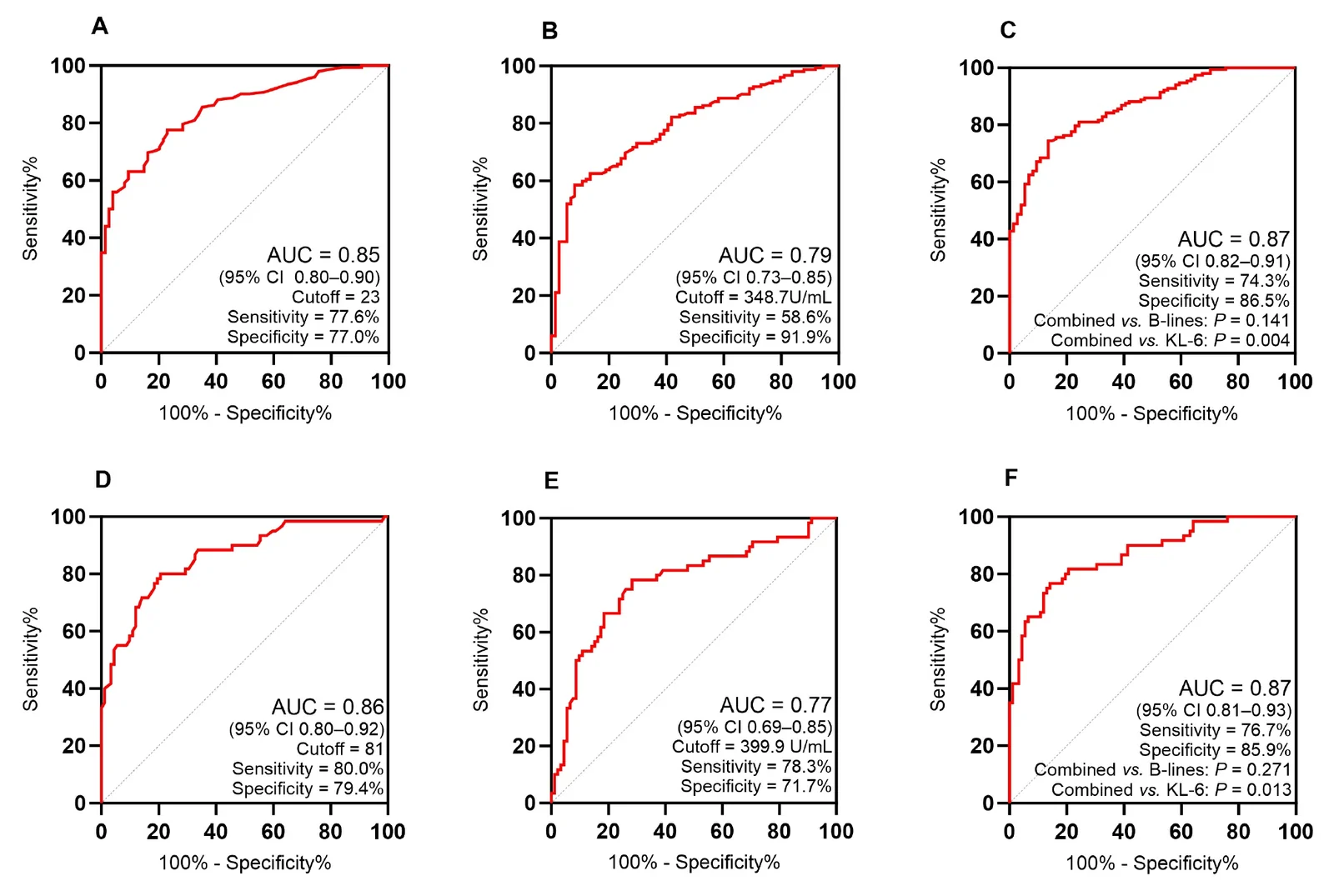
The area under the receiver operating characteristic (ROC) curves of (A) B-lines, (B) KL-6, and (C) the combined model for the discrimination between ILD and non-ILD, and (D) B-lines, (E) KL-6, and (F) the combined model for the discrimination between F-ILD and NF-ILD. AUC comparisons were performed using the DeLong test.

### Correlation with Radiological Findings

Correlation analysis revealed a moderate positive correlation between the number of B-lines and HRCT Warrick score (*r* = 0.61, *P* < 0.001; Figure 2A), suggesting that B-lines reflect the degree of radiological fibrosis. Furthermore, B-lines number demonstrated a moderate correlation with KL-6 levels (*r* = 0.48, *P* < 0.001; Figure 2B).

**Figure 2:**
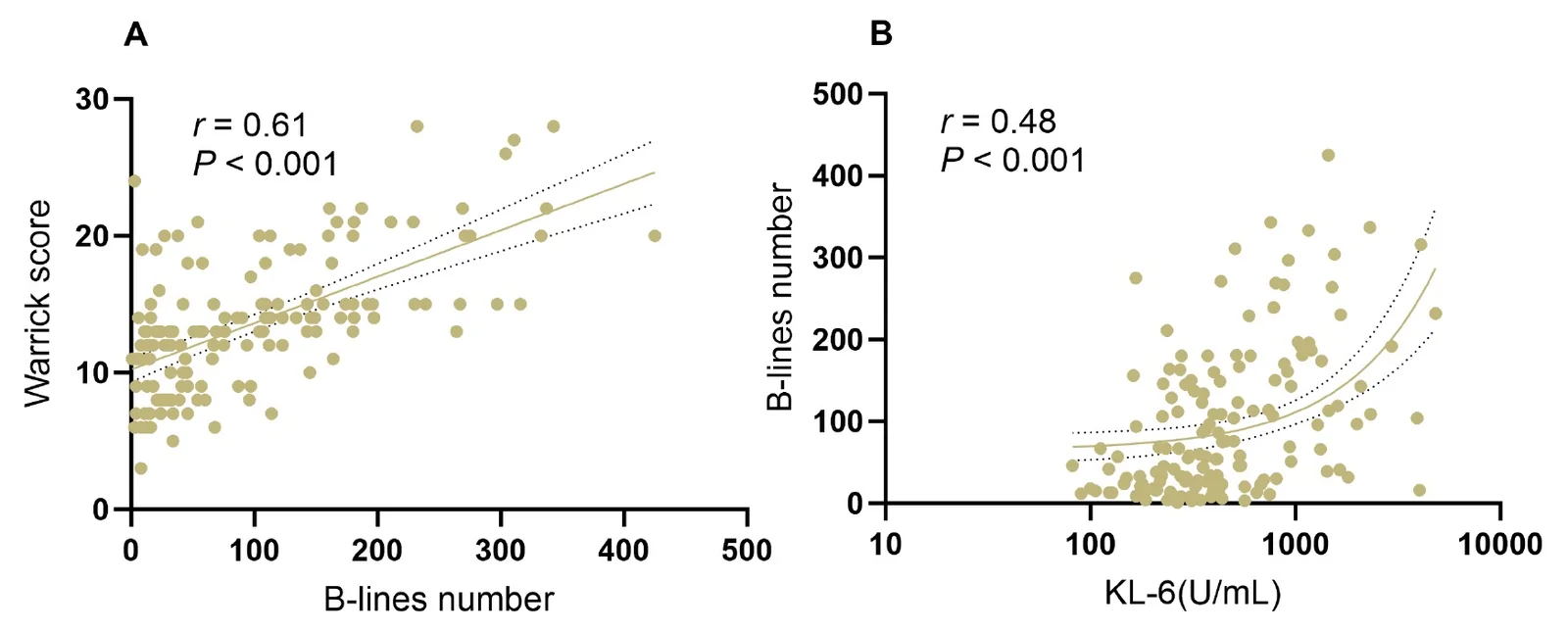
Scatter plots showing the correlations of (A) B-lines with HRCT Warrick score and (B) KL-6 with B-lines. Correlation coefficients (r) and P values are shown in ach panel. The x-axis in panel B is shown on a logarithmic scale for visualization; statistical analyses were performed using the original data.

### Discrimination of Fibrotic vs. Non-fibrotic ILD

Among the ILD cohort, those with fibrotic changes on HRCT exhibited significantly higher B-lines number and KL-6 levels than those with non-fibrotic disease (both *P* < 0.001; Table 2). ROC curve analysis indicated that B-lines could discriminate between fibrotic and non-fibrotic ILD with an AUC of 0.86 (95% CI: 0.80–0.92; Figure 1D). At an optimal cut-off value of 81, B-lines provided a sensitivity of 80.0% and a specificity of 79.4%. For KL-6, the AUC was 0.77 (95% CI: 0.69–0.85; Figure 1E), with an optimal cut-off of 399.9 U/mL yielding a sensitivity of 78.3% and a specificity of 71.7%. The combination of B-lines and KL-6 enhanced diagnostic performance, achieving an AUC of 0.87 (95% CI: 0.81–0.93; Figure 1F).

**Table 2:** Comparison of biomarkers between F-ILD and NF-ILD patients

Biomarkers	Fibrotic ILD (*n* = 60)	Non-fibrotic ILD (*n* = 92)	*P*
Warrick score, median (IQR)	15 (14–20)	12 (8–13)	<0.001
B-lines number, median (IQR)	161 (96–232)	33 (15–69)	<0.001
KL-6, U/mL, median (IQR)	770.0 (412.0–1316.3)	311.0 (223.3–426.7)	<0.001

ILD, interstitial lung disease; IQR, interquartile range; KL-6, Krebs von den Lungen-6.

### Discrimination of ILD Severity

According to the HRCT Warrick score, ILD was further categorized into mild (*n* = 16), moderate (*n* = 101), and severe (*n* = 35) subgroups. There was a statistically significant increase in the number of B-lines across increasing severity groups (15 [8–34] *vs*. 57 [26–123] *vs*. 161 [58–269], *P* < 0.001), indicating a strong correlation between lung ultrasound findings and HRCT severity. Serum KL-6 levels also demonstrated a significant increasing trend corresponding to disease severity (310.0 [212.2–419.7] *vs*. 374.0 [248.2–841.8] *vs*. 529.0 [322.0–915.0], *P* = 0.016). While KL-6 successfully differentiated between the extremes of disease severity (mild *vs*. severe, *P* = 0.014), its discriminatory capability was weak when comparing the mild and moderate subgroups specifically (*P* = 0.191; Table 3).

**Table 3:** Comparison of B-lines and KL-6 levels among ILD severity subgroup

Variable B-lines number, median (IQR)	Mild (*n* = 16) 15 (8–34)	Moderate (*n* = 101) 57 (26–123)	Severe (*n* = 35) 161 (58–269)	*P* <0.001	P^*^ Mild *vs*. Moderate (*P* = 0.004)
					Mild *vs*. Severe (*P* < 0.001)
					Moderate *vs*. Severe (*P* < 0.001)
KL-6, U/mL, median (IQR)	310.0 (212.2–419.7)	374.0 (248.2–841.8)	529.0 (322.0–915.0)	0.016	Mild *vs*. Moderate (*P* = 0.191)
					Mild *vs*. Severe (*P* = 0.014)
					Moderate *vs*. Severe (*P* = 0.207)

P* indicates adjusted *P* values after Bonferroni correction. ILD, interstitial lung disease; KL-6, Krebs von den Lungen-6; IQR, interquartile range.

## Discussion

The early identification and serial monitoring of CTD-ILD remain significant challenges in clinical rheumatology. While HRCT is the gold standard for morphological assessment, its use is limited by radiation exposure, cost, and limited accessibility for frequent follow-up. Our study reinforces the growing evidence that LUS B-lines and serum KL-6 are two significantly valuable, non-invasive and radiation-free biomarkers for CTD-ILD. Furthermore, our findings demonstrates that a combined approach using these biomarkers enhances diagnostic accuracy and their utility in distinguishing F-ILD.

The diagnostic performance of both B-lines (AUC = 0.85) and KL-6 (AUC = 0.79) in identifying CTD-ILD aligns with the existing body of literature.^[28, 29, 30, 31]^ The correlation we observed between B-lines and the HRCT Warrick score (*r* = 0.61, *P* < 0.001) is consistent with the strong correlation (*r =* 0.87, *P* < 0.001) reported in IIM-ILD.^[25]^ This confirms that B-lines, as a sonographic marker of interstitial syndrome, faithfully reflect the extent and severity of radiological involvement.

The moderate correlation between B-lines and KL-6 levels themselves (*r =* 0.48, *P* < 0.001) is a valuable finding, previously highlighted in a review on RA-ILD.^[18]^ This suggests that while these biomarkers capture related aspects of the disease process—B-lines indicating sonographic interstitial edema/fibrosis and KL-6 reflecting alveolar epithelial injury and regeneration—they provide complementary rather than redundant information.

A key feature of our study is the direct comparison between fibrotic and non-fibrotic ILD phenotypes. The significantly higher levels of both biomarkers in patients with fibrotic changes on HRCT underscores their potential role in phenotypic stratification, which has profound prognostic and therapeutic implications. The exceptional ability of B-lines to discriminate between these groups (AUC = 0.86), superior to that of KL-6 (AUC = 0.77), suggests that LUS may be particularly sensitive to the structural alterations characteristic of fibrosis. This finding is of high clinical relevance, as identifying a fibrotic phenotype is often a trigger for initiating or intensifying antifibrotic therapy.

An significant finding from our work is the consistent additive value of combining B-lines and KL-6. The integrated model achieved the highest AUC for both overall CTD-ILD diagnosis (AUC = 0.87) and fibrotic phenotype discrimination (AUC = 0.87). This synergistic effect can be explained by their different pathophysiological correlates. KL-6, as a specific serum marker, offers high specificity (91.9% in our cohort for diagnosis), making it an excellent „rule-in“ tool. LUS B-lines, with their high sensitivity and dynamic range, provide a direct, real-time assessment of lung involvement. This combination effectively mitigates the individual limitations of each test: the potential for false-positive B-lines in non-ILD conditions like cardiogenic pulmonary edema, and the lower sensitivity of KL-6 in some early or mild cases. Our results are supported by the recent nomogram study by Zhang *et al*.,^[32]^ which demonstrated that adding B-lines and KL-6 to a clinical model dramatically increased the predictive power for IIM-ILD (AUC from 0.876 to 0.951). Similarly, the case-control study on RAILD proposed a combined cut-off strategy, affirming the clinical utility of this dual-biomarker approach.^[33]^

Both B-lines and KL-6 increased significantly with ILD severity. B-lines showed a significant, stepwise progression across mild, moderate, and severe groups, proving highly sensitive for assessing disease extent. While KL-6 confirmed an overall upward trend, it could not reliably distinguish mild from moderate disease. This suggests KL-6 requires a threshold of damage to rise, making it better for identifying advanced disease rather than early-stage discrimination.

Our study has several limitations, including its single-center and retrospective design and relatively small sample size. In addition, LUS is inherent operator-dependency although this can be mitigated with standardized protocols. Therefore, our findings should be interpreted with caution. Future large-scale, multi-center, prospective longitudinal studies are needed to validate the proposed cut-off values. Most importantly, research should investigate whether serial changes in this combined biomarker score can reliably predict disease progression, treatment response.

## Conclusions

In conclusion, our findings support the use of LUS B-lines and serum KL-6 as potential non-invasive, radiation-free biomarkers for managing CTD-ILD. The synergistic effect of their combination offers a novel strategy that can be integrated into routine clinical practice for comprehensively assessment of CTD-ILD.
